# Phenomenological Modelling of COVID-19 Epidemics in Sri Lanka, Italy, the United States, and Hebei Province of China

**DOI:** 10.1155/2020/6397063

**Published:** 2020-10-18

**Authors:** A. M. C. H. Attanayake, S. S. N. Perera, S. Jayasinghe

**Affiliations:** ^1^Department of Statistics & Computer Science, Faculty of Science, University of Kelaniya, Sri Lanka; ^2^Research & Development Centre for Mathematical Modelling, Department of Mathematics, Faculty of Science, University of Colombo, Sri Lanka; ^3^Department of Clinical Medicine, Faculty of Medicine, University of Colombo, Sri Lanka

## Abstract

The COVID-19 pandemic has resulted in increasing number of infections and deaths every day. Lack of specialized treatments for the disease demands preventive measures based on statistical/mathematical models. The analysis of epidemiological curve fitting, on number of daily infections across affected countries, provides useful insights on the characteristics of the epidemic. A variety of phenomenological models are available to capture the dynamics of disease spread and growth. The number of daily new infections and cumulative number of infections in COVID-19 over four selected countries, namely, Sri Lanka, Italy, the United States, and Hebei province of China, from the first day of appearance of cases to 2^nd^ July 2020 were used in the study. Gompertz, logistic, Weibull, and exponential growth curves were fitted on the cumulative number of infections across countries. AIC, BIC, RMSE, and *R*^2^ were used to determine the best fitting curve for each country. Results revealed that the most appropriate growth curves for Sri Lanka, Italy, the United States, and China (Hebei) are the logistic, Gompertz, Weibull, and Gompertz curves, respectively. Country-wise, overall growth rate, final epidemic size, and short-term forecasts were evaluated using the selected model. Daily log incidences in each country were regressed before and after the identified peak time of the respective outbreak of epidemic. Hence, doubling time/halving time together with daily growth rates and predictions was estimated. Findings and relevant interpretations demonstrate that the outbreak seems to be extinct in Hebei, China, whereas further transmissions are possible in the United States. In Italy and Sri Lanka, current outbreaks transmit in a decreasing rate.

## 1. Introduction

COVID-19 or coronavirus disease 2019 is a pandemic which initiated in Wuhan, China, in December 2019. At present (02/07/2020), the pandemic has recorded a total number of 10,533,779 infections and 512,842 deaths in the world [1]. Daily infections and deaths are increasing at an alarming rate. World Health Organization (WHO) estimated 85,263 total confirmed cases and 4,648 deaths in China [1]. The number of infections for Italy, Sri Lanka, and the United States were 240,760, 2,054, and 2,616,949, and deaths were 34,788, 11, and 127,133, respectively. On 19^th^ March 2020, Italy was the country where the highest number of deaths was recorded in the world. Since then, Italy has attracted the attention of the public on its active engagement with the coronavirus disease 2019. In Sri Lanka, the first infection was identified on 11^th^ March 2020. Afterwards, as with other countries, several control measures such as isolation of cases, quarantine of suspects, lockdown of the country or permitting island-wide curfew, limitation of public gatherings, annunciation of social distancing, increase awareness of the disease through media, and encouraging people to stay in their homes were implemented. Clearly, China was at the ending phase of the epidemic when Italy entered into the phase of a growing epidemic in February 2020 and is now passed its middle phase. Sri Lanka had the beginnings of the epidemic in March 2020 and is moving to its ending phase. The United States entered into the battle of the epidemic in January 2020. As of 2^nd^ July 2020, it is the country which reported the highest number of reported cases in the world and illustrated rapid spread of the disease in the near future. Since these countries are in different phases of the epidemic, we decided to analyze them further and draw some conclusions.

In order to mitigate the epidemic, control measures should be coupled with mathematical and statistical modelling to fine tune strategies by anticipating the dynamics of disease spread. Applications of phenomenological models in describing a growth of natural phenomenon can be found in the literature [2–5]. Among several phenomenological models, logistic, Gompertz, exponential, and Weibull growth models are appropriate in modelling the growth of an epidemic. These models are able to capture empirical patterns of an epidemic and are useful in predicting dynamics of the disease. Prediction of models will be beneficial for health care professionals to allocate necessary resources and implement preventive measures in a timely manner. The aim of this study was to compare the logistic, Gompertz, exponential, and Weibull growth models to determine which best fits the data of coronavirus disease 2019 in different countries. Further, the epidemics in each of the selected countries were described and predicted by examining transmission potential of the disease.

The analysis of epidemiological curve fitting on the number of daily infections provides a useful insight on determining the variety of statistical features of an outbreak [6, 7]. To capture the epidemic pattern, one can fit a regression model on log incidences over time. Once the peak of the distribution has been identified, pre- and post peak models of the distribution assist to identify the point of zero incidences of an outbreak. Further, the estimation of doubling time or halving time together with daily growth rates and predictions was obtained from the curve fitting.

In this study, Gompertz, logistic, Weibull, and exponential growth curves are fitted on the cumulative number of infections over the selected four countries, namely, Sri Lanka, Italy, the United States, and Hebei province of China. The best fitted growth curve was identified for each country in order to compute the dynamics and generate short-term predictions of the disease. Further, useful characteristics of epidemiological curves were extracted and predictions were generated using pre- and post peak models.

## 2. Materials and Methods

### 2.1. Data

Daily accumulated coronavirus disease 2019 cases were downloaded [8] for the four countries from the first day of appearance of cases to 2^nd^ July 2020. Data recording began in Hebei province of China on 22^nd^ of January 2020 and Italy on 31^st^ of January 2020. Sri Lanka was on 10^th^ of March 2020 and the United States on 24^th^ January 2020. Daily new infections were calculated from the downloaded data.

### 2.2. Methods and Definitions

#### 2.2.1. Mathematical Models

Four different nonlinear growth models were used to pinpoint the epidemic curves of coronavirus disease 2019 over four countries: Sri Lanka, Italy, the United States, and Hebei province of China. The four models used are logistic, Gompertz, exponential, and Weibull growth models. These simple models are widely used in modelling cumulative growth processes [9–11]. Different nonlinear growth models have different characteristics [10], and therefore, the determination of the best model that fits on a growth process is important. R software is used for the analysis [12]. The models were fitted using functions encapsulated in “drc” and “incidence” packages available with the software [13, 14].

*(1) Exponential Growth Model.* Exponential growth models are suitable in fitting the early phase of an epidemic because it is not realistic to represent a natural growth by an exponential model which may never end. An exponential growth is explained as [3]

(1) $$\begin{array}{c} \frac{dN}{dt}=rN, \end{array}$$

with the solution

(2) $$\begin{array}{c} N\left(t\right)=N_{0}e^{rt}, \end{array}$$

where *N*_0_ denotes the size of the population at time zero, *N* denotes the size of the population, *t* denotes time, and *r* is the per capita rate of increase.

*(2) Logistic Growth Model.* Logistic growth models have an approximately exponential nature at the first phase and continue the growth at a reduced rate of growth where it finally reaches its maximum [4]. The mathematical model that explains the logistic growth is expressed as

(3) $$\begin{array}{c} \frac{dN}{dt}=rN\left(\frac{K-N}{K}\right), \end{array}$$

where *N* denotes the size of the population, *t* denotes time, *K* is the carrying capacity, and *r* is the per capita growth rate.

The solution of (3) is given:

(4) $$\begin{array}{c} N\left(t\right)=\frac{K}{1+a\exp\left(-rt\right)}, \end{array}$$

where *K*/(1 + *a*) is the initial value.

*(3) Gompertz Growth Model.* The Gompertz model is a sigmoid function which describes the slowest rate of growth at the beginning and at the end [9]. The model can be written as

(5) $$\begin{array}{c} N\left(t\right)=N\left(0\right)\exp\left(-c\left(\exp\left(at\right)-1\right)\right), \end{array}$$

where *a* is the rate of growth, *N*(0) is the initial number of organisms, *c* denotes the displacement across the *x*-axis, and *b* and *c* are positive numbers.

*(4) Weibull Growth Model.* The four-parameter Weibull model is one of the nonlinear growth models available to model biological processes. The model can be written as

(6) $$\begin{array}{c} N\left(t\right)=\beta_{0}-\beta_{1}\exp\left(-\beta_{2}t^{\beta_{3}}\right), \end{array}$$

where *β*_0_ is the largest growth size, *β*_1_ is the scale parameter related to the initial size, *β*_2_ is the relative growth size, and *β*_3_ is the shape parameter [11].

#### 2.2.2. Akaike Information Criterion (AIC) and Bayesian Information Criterion (BIC)

AIC measures the relative distance among the true likelihood function of the original data series and the built-in likelihood function of the model. AIC is calculated by using the formula

(7) $$\begin{array}{c} \text{AIC}=n\;\ln\left(\frac{\text{SSE}}{n}\right)+2k, \end{array}$$

where *k* is the number of parameters in the model, *n* is the number of observations, and SSE is the sum of squared errors of the model.

BIC is another criterion used to select the best model. It is based on a Bayesian framework and measures the posterior probability of a model being true. The following equation is used to estimate the BIC of a model:

(8) $$\begin{array}{c} \text{BIC}=n\;\ln\left(\frac{\text{SSE}}{n}\right)+k\ln\left(n\right). \end{array}$$

The parameters have the same meaning as in AIC. A model with the minimum AIC or BIC value will be the best model [3]. Both AIC and BIC are approximately accurate, and the relative power of the two measures depends with the study design [15].

#### 2.2.3. Coefficient of Determination (*R*^2^) and Residual Mean Squared Error (RMSE)

The *R*^2^ value is calculated from the formula

(9) $$\begin{array}{c} R^{2}=1-\frac{\text{SSE}}{\text{SST}}, \end{array}$$

where SSE is the error sum of squares of the model and SST is the total sum of squares of the model. Therefore, *R*^2^ explains the variance of the model relative to the total variance. When the two variances are perfectly correlated, there is no variance at all. If the *R*^2^ is close to 100%, then the model is acceptable.

The RMSE is the average of the squared errors of the residuals of a model. It is calculated as follows:

(10) $$\begin{array}{c} \text{RMSE}=\sqrt{\text{MSE}}=\sqrt{\text{SSE}}, \end{array}$$

where SSE is the error sum of squares of the model. The lower the value of RMSE indicates a better model [3]. The *R*^2^ and RMSE are closely related and widely used as accuracy measures.

#### 2.2.4. Epidemic Curve

The epidemiological curve or an epidemic curve is a curve use in epidemiology to extract meaningful characteristics of an epidemic [9, 10]. In particular, it shows the transmission of the disease over time. Further, disease outliers, magnitude of the epidemic, its peak time, applicable trends, and incubation period are identified by the curve.

#### 2.2.5. Growth Rate

Growth rate denotes the change of a particular variable over a specified time duration. This statistic is useful to describe the performance of a variable as well as to predict the performance. The growth rate is calculated as follows:

(11) $$\begin{array}{c} \text{Daily growth rate}=\frac{\text{Present value of the variable}-\text{Past value of the variable}}{\text{Past value of the variable}}. \end{array}$$

The growth rate over *n* time duration is given by

(12) $$\begin{array}{c} \text{Average growth rate}={\left(\frac{\text{Present value of the variable}}{\text{Past value of the variable}}\right)}^{1/n}-1. \end{array}$$

## 3. Results and Discussion

**Figure 1:**
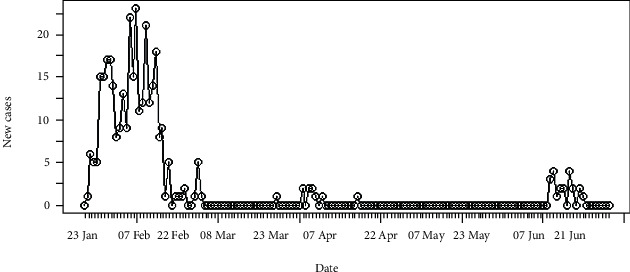
Daily incidences of coronavirus in Hebei.

**Figure 2:**
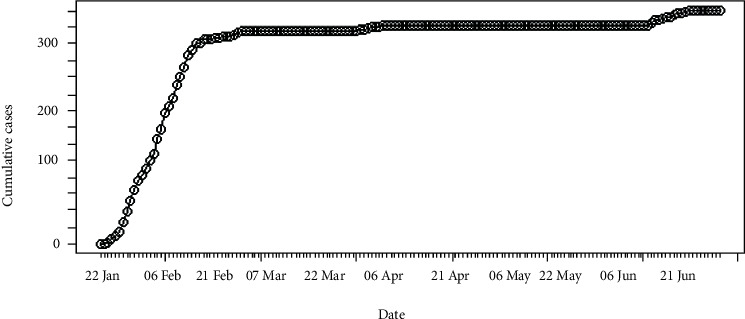
Cumulative incidences of coronavirus in Hebei.

The number of daily incidences of coronavirus disease 2019 in Hebei province of China is shown in Figure 1 and accumulated incidences in Figure 2. In between January and March of 2020, the number of daily incidences varies with the maximum value of 23 cases. The figures illustrate that after February of 2020, the number of reported incidences was lower in Hebei and the epidemic reaches its saturation. Around 14^th^ June of 2020, few novel cases were reported, and as of 2^nd^ July 2020, reported cases were at a minimum level.

**Figure 3:**
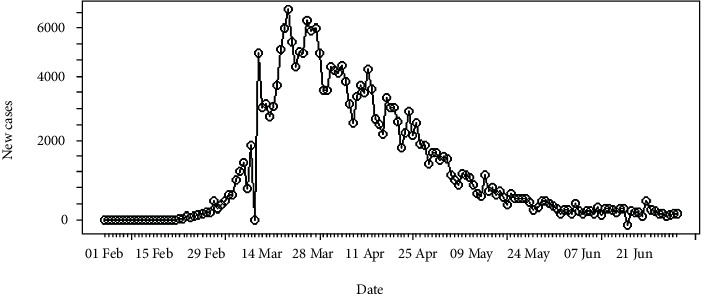
Daily incidences of coronavirus in Italy.

**Figure 4:**
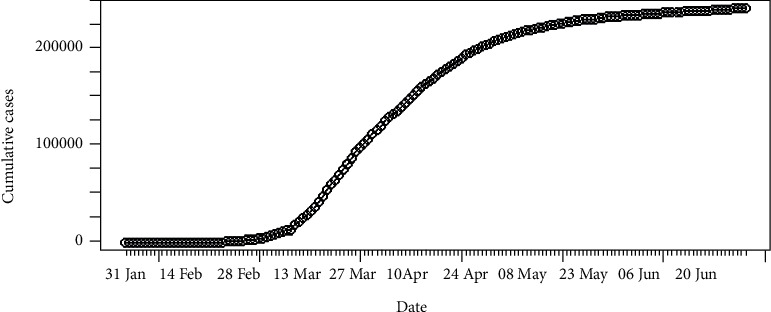
Cumulative incidences of coronavirus in Italy.

The daily number of new infections in Italy is depicted in Figure 3 and its cumulative distribution in Figure 4. Slow transmission is apparent at the beginning stage of the epidemic. The daily incidences in Italy range in between 0 and 6,557 cases which has a mean of 1,577 cases with standard error of mean of 147. According to Figure 4, the epidemic is downgrading but still has not reached its saturation. Therefore, more cases can be expected under the reduced rate.

**Figure 5:**
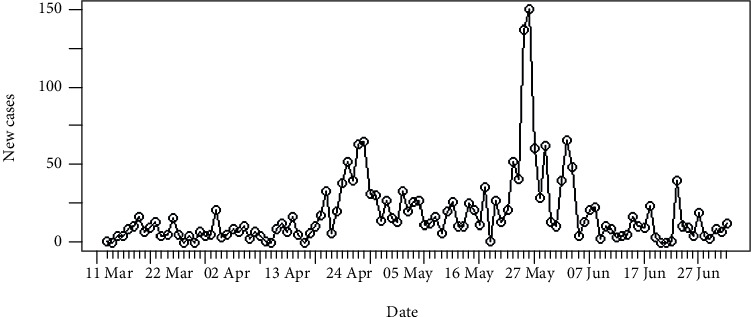
Daily incidences of coronavirus in Sri Lanka.

**Figure 6:**
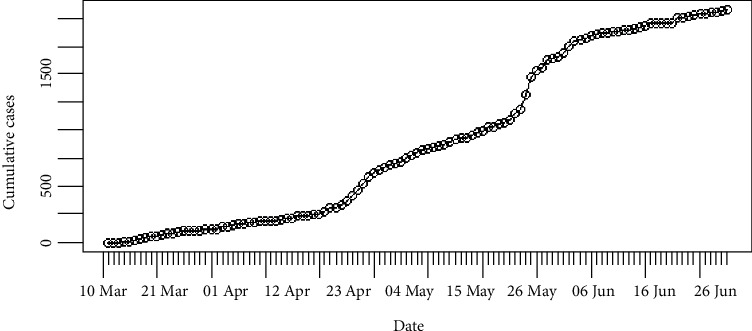
Cumulative incidences of coronavirus in Sri Lanka.

In Sri Lanka, the number of daily recordings of coronavirus disease 2019 is represented in Figure 5. The highest number of recording was reported on the 27^th^ of May 2020 which was 150 cases. The cumulative number of infections in Sri Lanka is shown in Figure 6. It is apparent that the epidemic is still progressing slowly in Sri Lanka as of 2^nd^ of July 2020.

**Figure 7:**
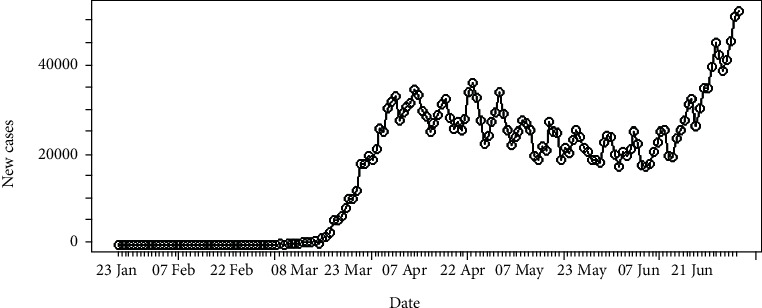
Daily incidences of coronavirus in the United States.

**Figure 8:**
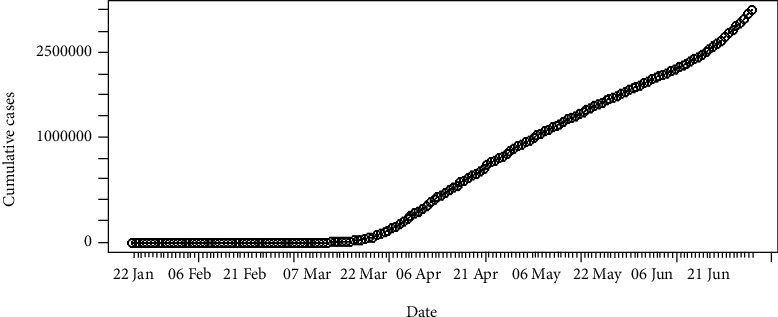
Cumulative incidences of coronavirus in the United States.

The daily number of new infections in the United States and its cumulative distribution are shown in Figures 7 and 8, respectively. The pattern of the epidemiological curve is undulating, and it is apparent that the number of reported cases increased drastically as of 2^nd^ July 2020. The epidemic seems to be spreading further stressing precautions to mitigate the impact of the disease.

**Figure 9:**
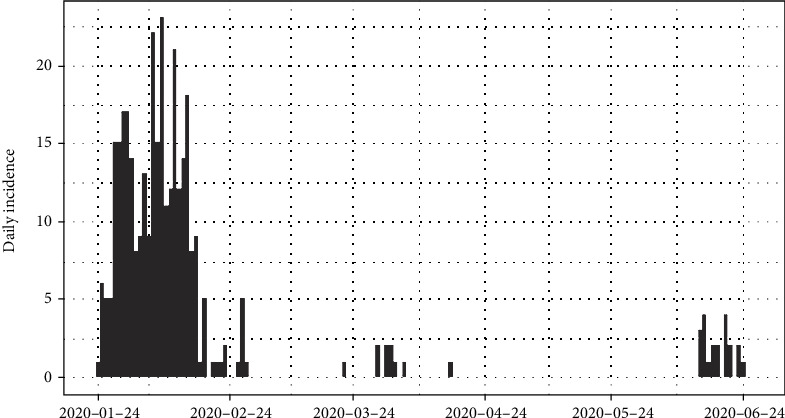
Epidemic curve of coronavirus disease 2019 in Hebei province, China.

The epidemiological curve for Hebei, China, in Figure 9 shows that the number of cases increased till mid of February 2020 and then progressively decreased. This was an indication that the peak time of the outbreak in China had been reached on 8^th^ February 2020.

**Figure 10:**
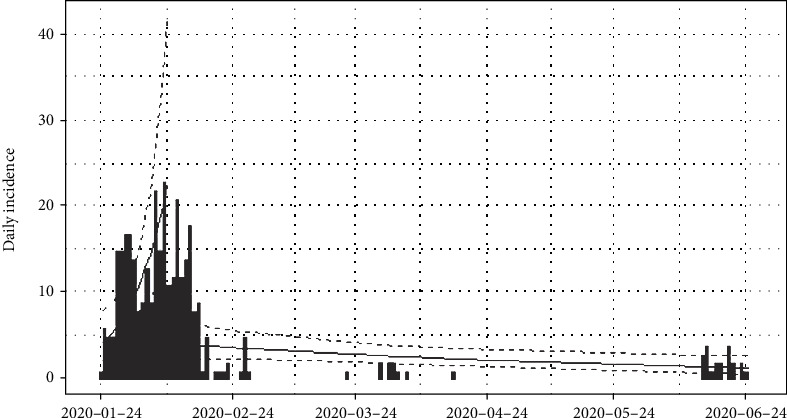
Prediction models for infections in Hebei province, China.

We then divided the entire data period into two halves as before and after the peak point and regressed log incidences over time for each split. The fitted models are exponential curves as depicted in Figure 10. Prior to 8^th^ February 2020, the number of incidences was increased at the rate of doubling in every 6 days (95% CI [3.86, 17.49]), and the daily growth rate was estimated as 0.11 with 95% confidence interval (0.04, 0.18). According to the fitted log-linear model, after the 8^th^ February 2020, the reported incidences were downgraded at the rate of halving in every 89 days (95% CI [47.71, 688.88]) with a daily decreasing rate of -0.008 (95% CI [-0.015, -0.001]). Predictions of the post peak log-linear model indicated that the epidemic almost reached to zero point of infections.

The household-based outdoor restrictions were started in Hebei province of China in 7^th^ February 2020 [16]. The point is very close to the identified peak point of the curve. Therefore, the post peak model is in line with the government decisions, and the reduction of growth rate can be justified.

**Figure 11:**
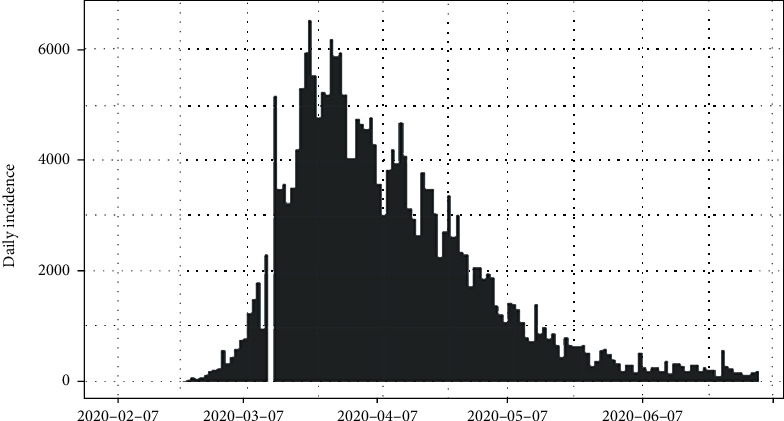
Epidemic curve of coronavirus disease 2019 in Italy.

**Figure 12:**
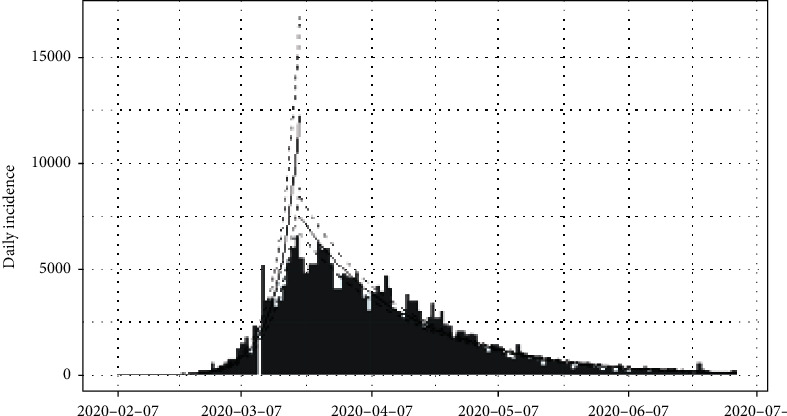
Prediction models for infections in Italy.

The epidemiological curve for Italy is shown in Figure 11. It can be concluded that the peak time of the outbreak in Italy had been reached as per apparent growth and shrinkage patterns of the epidemiological curve. The estimated peak time was on 21^st^ March 2020, and the pre- and post peak data were separated and each split was regressed by log incidences over time. The fitted exponential curves are shown in Figure 12. From the first date of appearance of cases to 21^st^ March 2020, the number of COVID-19 infections increased at the rate of doubling in every 4 days (95% CI [3.27, 3.92]). The daily growth rate was estimated as 0.19 (95% CI [0.18, 0.21]). After the 21^st^ March 2020, the reported COVID-19 infection rate decreased more slowly, halving every 18 days (95% CI [16.96, 18.72]). The daily decreasing rate was -0.04 (95% CI [-0.041,-0.037]). Predictions of the post peak log-linear model indicated that the COVID-19 epidemic in Italy is progressing with a decreasing rate.

The first lockdown in Italy began around 21^st^ February 2020, and several lockdowns continued and expanded from that point [17]. On 21^st^ March 2020, the Italian Prime Minister announced a further enlargement of the lockdown and asked for a generalized shutdown of the Italian production system [17]. These decisions seem to have had an effect on reducing recordings of the number of infections with coronavirus.

**Figure 13:**
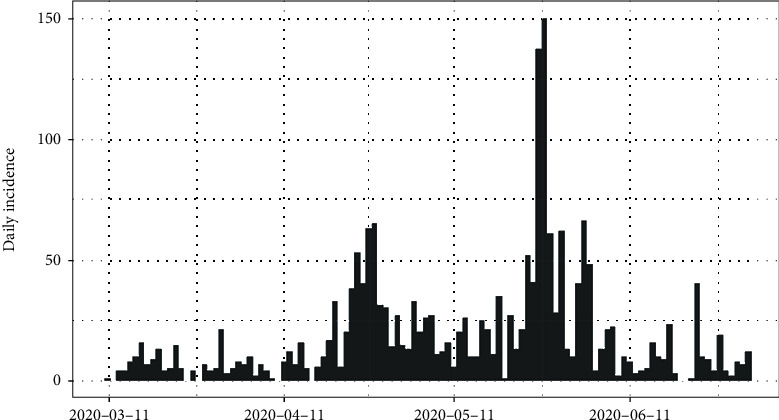
Epidemic curve of coronavirus disease 2019 in Sri Lanka.

**Figure 14:**
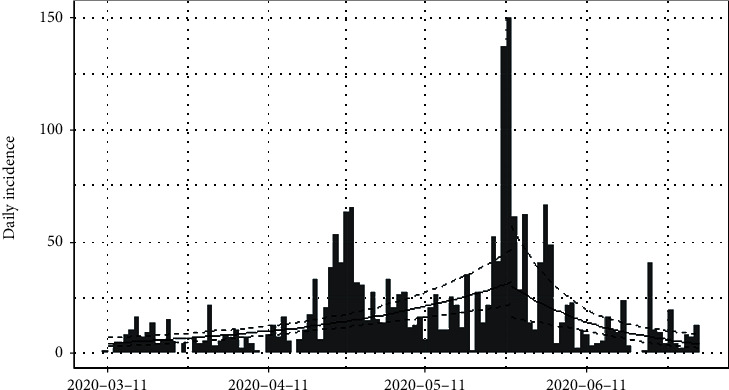
Prediction models for infections in Sri Lanka.

Figure 13 shows the epidemiological curve for Sri Lanka. Clearly, Sri Lanka reached its maximum on 27^th^ May 2020. Prior to 27^th^ May 2020, the number of incidences was increased at the rate of doubling in every 27 days (95% CI [20.10, 41.36]), and the daily growth rate was estimated as 0.02 with 95% confidence interval (0.01, 0.03). According to the post peak log-linear model, the reported incidences were downgraded at the rate of halving in every 13 days (95% CI [8.07, 29.53]) with a daily decreasing rate of -0.05 (95% CI [-0.09, -0.02]). Predictions of the fitted log-linear model indicated that the epidemic is progressing in Sri Lanka in a decreasing rate. Prediction models are shown in Figure 14.

On 20^th^ March 2020, the Sri Lankan President announced a lockdown-styled curfew for the entire nation during several hours of the day, and from 24^th^ March 2020, the government imposed curfew for very high-risk zones such as Colombo, Gampaha and Kalutara districts [18]. Sri Lanka took immediate actions in controlling the disease from the first case of reporting of the epidemic and hence reached the ending phase of the epidemic shortly.

**Figure 15:**
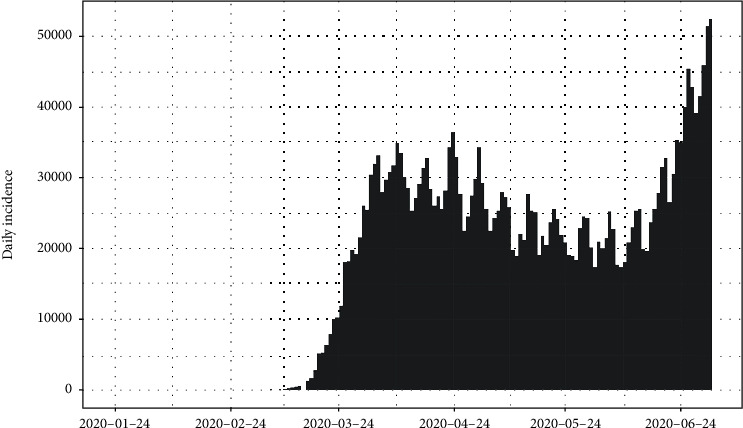
Epidemic curve of coronavirus disease 2019 in the United States.

**Figure 16:**
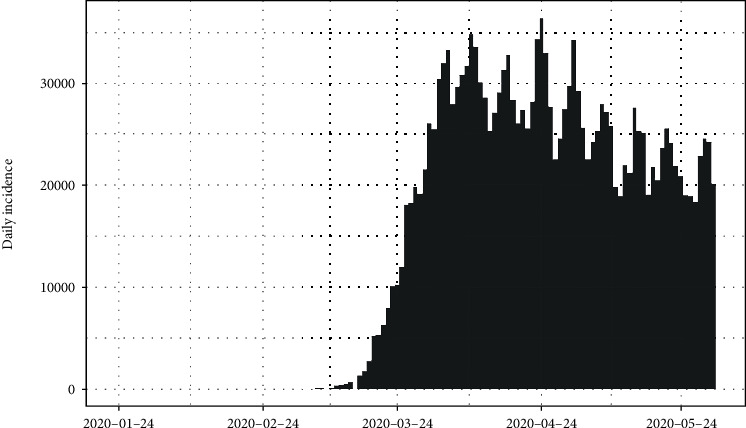
Epidemic curve for the first group of data.

**Figure 17:**
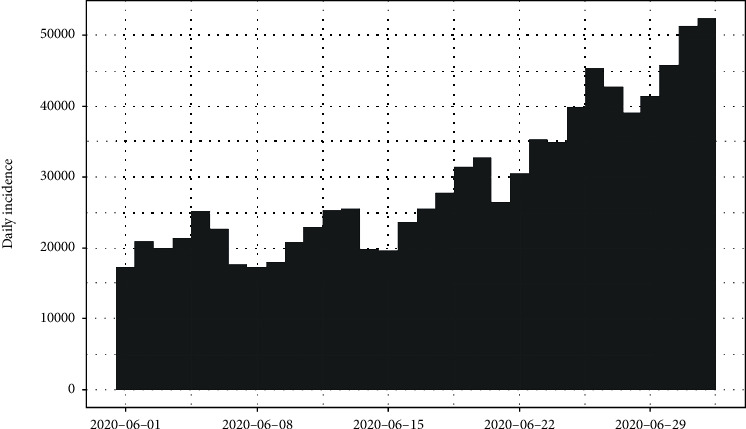
Epidemic curve for the second group of data.

The epidemiological curve for the United States is represented in Figure 15. The figure shows that the number of cases increased till 24^th^ April 2020 and then slowly decreased around 31^st^ May 2020. Then, again the reported cases progressively increased up to the last date of data gathering of this study (02/07/2020). Therefore, the data set is divided into two groups as from the first day of appearing of cases to the 31^st^ of May 2020 as the first group and the second group as from the 1^st^ of June 2020 to the 2^nd^ of July 2020. The epidemiological curves for divided data sets are shown in Figures 16 and 17.

**Figure 18:**
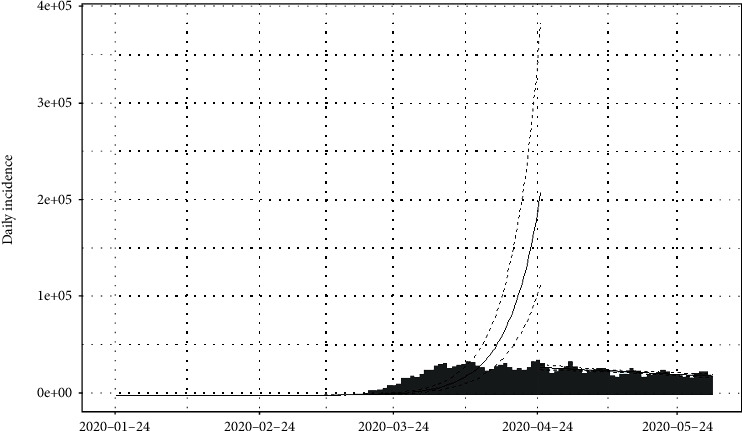
Prediction models for infections in the United States—first data set.

The estimated peak time was on 24^th^ April 2020 in the first group of the data set. Prior to the estimated peak time in the first data set, the number of incidences was increased at the rate of doubling in every 5 days (95% CI [4.26, 5.20]) and the daily growth rate was estimated as 0.15 with 95% confidence interval (0.13, 0.16). According to the post peak log-linear model, the reported incidences were downgraded at the rate of halving in every 71 days (95% CI [50.5, 119.5]) with a daily decreasing rate of -0.009 (95% CI, [-0.01, -0.006]). Pre- and post prediction models are shown in Figure 18.

**Figure 19:**
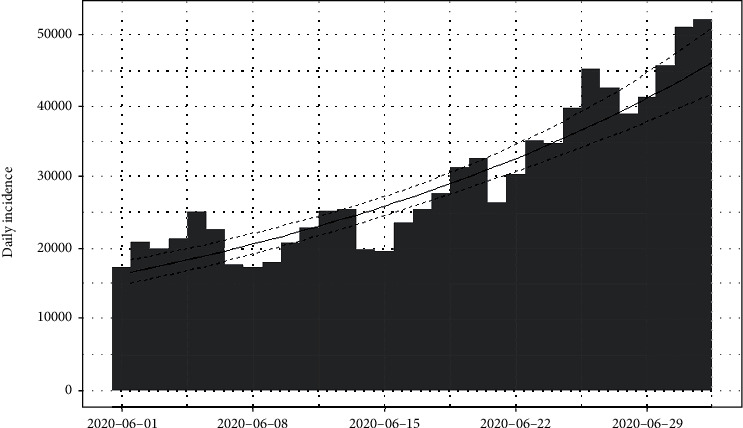
Prediction model for infections in the United States—second data set.

For the second group of the data set, the number of incidences was increased at the rate of doubling in every 21 days (95% CI [18.04, 25.25]), and the daily growth rate was estimated as 0.03 with 95% confidence interval (0.02, 0.038). The prediction model is shown in Figure 19.

In the United States, lockdown periods varied across different states and the epidemic is further spreading with a large number of reported cases.

Daily growth rates of each of the countries from the pre- and post peak models are summarized in Table 1.

According to Table 1, it can be concluded that within the prepeak models, the highest daily growth exhibits in Italy whereas the lowest is in Sri Lanka. Therefore, the number of daily infections with coronavirus disease 2019 was highest in Italy than in the other three countries before their respective peaks. A sharp increase (higher slope) in the epidemic curve of the prepeak model is apparent in Italy. Although the number of daily infections was relatively lower in Sri Lanka before the peak point, after the peak point, the number of daily infections was highest than in the other three countries. After the peak point, the lowest number of daily infections was reported in China. Therefore, the restrictions/lockdowns implemented by the government of China after the peak point were efficient in reducing the number of daily reported cases with coronavirus disease 2019.

**Figure 20:**
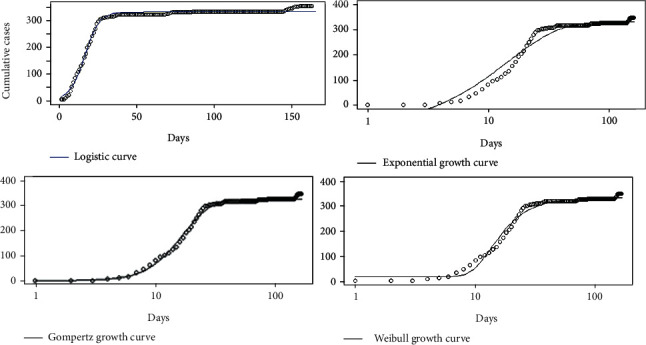
Growth models for Hebei province of China.

Daily accumulations of coronavirus disease 2019 were analyzed using phenomenological models for the countries Sri Lanka, Italy, the United States, and Hebei province of China. In particular, Gompertz, logistic, exponential, and Weibull growth curves were fitted on the cumulative number of infections over the four countries, and the best fitted growth curve was identified for each country. The best fitted growth curve had the minimum AIC, BIC, and RMSE values and the highest *R*^2^ value. The fitted growth models for Hebei province of China are given in Figure 20.

**Figure 21:**
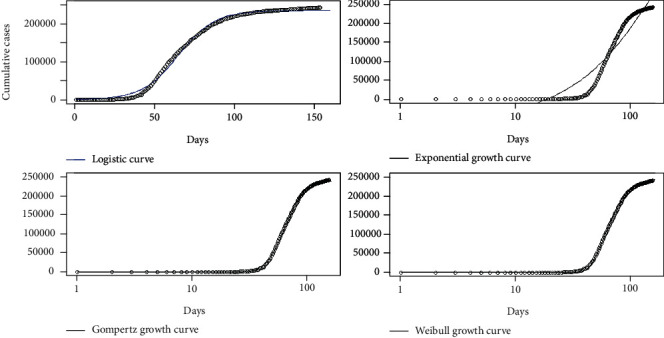
Growth models for Italy.

**Figure 22:**
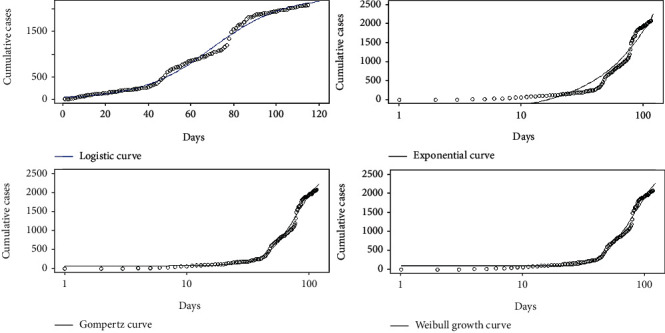
Growth models for Sri Lanka.

**Figure 23:**
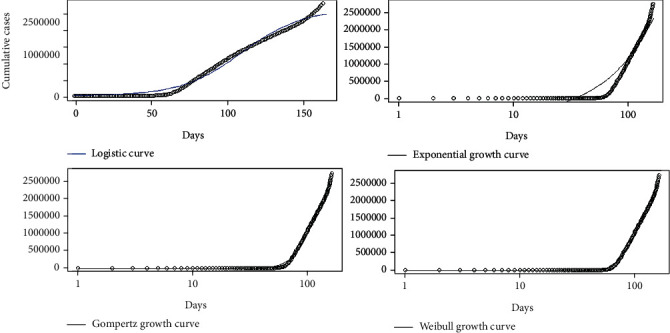
Growth models for the United States.

The fitted growth models for Italy, Sri Lanka, and the United States are given in Figures 21–23, respectively.

The results of goodness of fit statistics for each of the growth model in each country are summarized in Table 2.

The lower the AIC, BIC, and RMSE values and the higher the *R*^2^ value reflect the quality of the model.

Results revealed that the most appropriate growth curves for Sri Lanka, Italy, the United States, and China (Hebei) are the logistic, Gompertz, Weibull, and Gompertz curves, respectively. The overall growth rate estimated from the logistic growth model for Sri Lanka was 16. The final epidemic size was estimated as 2344 cases. Short-term predictions from the logistic growth curve revealed that the epidemic will reach close to zero in the near future. The Gompertz model fitted on the data of Italy revealed that when the days increase, the number of coronavirus infections decreases at a rate of 0.057. The upper asymptote value (the maximum value approached by the curve) was estimated as 249,290 cases. Short-term predictions from the Gompertz growth curve revealed that the coronavirus outbreak was progressing at a decreasing rate in Italy. The best growth model fitted on the data of Hebei province of China estimated that when the days increase, the number of coronavirus infections decreases at a rate of 0.14. The upper asymptote value was estimated as 358 cases. Short-term predictions from the Gompertz growth curve revealed that the coronavirus outbreak is in control in Hebei province of China. For the United States, estimated upper asymptote value was 4,990,200. Further, the overall growth rate estimated from the Weibull growth model was 12. Short-term predictions from the growth model revealed that the coronavirus outbreak is increasing further in the United States.

All the countries under study except the United States are near or have reached the ending of the epidemic, and the lowest upper asymptote value was estimated for the Hebei province in China.

## 4. Conclusion

This study successfully models the ongoing coronavirus disease 2019 outbreak present in Hebei province of China, Italy, the United States, and Sri Lanka using phenomenological modelling and epidemiological curve fitting analysis for the period of date of appearance of cases in each country to the 2^nd^ July 2020. The possibility of modelling the outbreak through four growth models, logistic, exponential, Gompertz, and Weibull, were tested. Findings disclosed that the most appropriate growth curves for Sri Lanka, Italy, the United States, and China (Hebei) are the logistic, Gompertz, Weibull, and Gompertz curves, respectively. The epidemiological curves of each country were analyzed to extract useful characteristics of the coronavirus outbreak. Prepeak and post peak log-linear models were fitted on the epidemic curve to predict the spread of coronavirus disease 2019 infection in the future. By comparing growth rates of the prepeak models of selected countries, it can be concluded that the highest growth rate of daily infections with coronavirus disease 2019 was reported in Italy. Hebei province records the lowest growth rate in daily infections during the post peak period. Therefore, the restrictions/lockdowns implemented by the government of China during the peak period were efficient and successful in reducing the number of daily reported cases with coronavirus disease 2019. In Sri Lanka, the post peak growth was higher than the other three countries suggesting that the country should enhance its screening facilities to detect the infections at the early stage. Daily growth of 3% infections in the United States with the current data indicates that the coronavirus disease 2019 is spreading further within the country.

The results show that the epidemic seems extinct in Hebei, China, whereas further transmissions are possible in the United States. Therefore, effective controlling measures need to be continued and enhanced with careful supervision in order to keep the epidemic under control in the United States. In Italy and Sri Lanka, the current outbreaks are transmitting in a decreasing rate. Hence, controlling mechanisms seem to be on track and should be continued to eradicate the infection.

The analysis of this study was done assuming that all the cases were reported accurately. However, there may be underreporting of cases and inadequate screening which lead to a disparity between actual values and the predicted values [19, 20]. Further, sudden and unexpected recordings may occur in countries that cannot be modelled and predicted by the fitted models. In particular, sudden increases are possible with countries which are engaged in reactive testing strategies rather than proactive aggressive testing strategies. Some countries do not conduct enough number of testing to cover larger percentages of the population which results in unexpected spikes in the recordings. Some countries may change their regulations for returning nonresident citizens. This may lead to sudden deviations in daily incidents due to imported cases. These types of uncertainties always limit the effectiveness and productivity of a model which deals with the dynamics of an epidemic like novel coronavirus.

Findings of this study will be beneficial for health care professionals to allocate necessary resources efficiently and effectively. Further, results of this study guide authorities to take appropriate measures in social distancing and regulations. There is a possibility of applying the same methodology used in this study for other countries which have been affected by the coronavirus disease 2019 outbreak, to understand/control the epidemic.

## Figures and Tables

**Table 1:** Daily growth rates of the countries.

Country	Prepeak model	Post peak model
Italy	0.19	-0.04
Sri Lanka	0.02	-0.05
China	0.11	-0.008
United States (for group 1)	0.15	-0.009

**Table 2:** Goodness of fit statistics of growth models.

Country	Model	*R* ^2^	AIC	BIC	RMSE
Sri Lanka	Logistic	99.1	1311	1322	70
	Exponential	95	1509	1520	165
	Gompertz	98.9	1339	1352	78
	Weibull	98.7	1360	1374	86

Italy	Logistic	99.6	3128	3141	6077
	Exponential	92.7	3584	3596	26688
	Gompertz	99.9	2738	2753	1697
	Weibull	99.9	2764	2779	1849

China (Hebei)	Logistic	98.9	1163	1175	8
	Exponential	96.2	1376	1389	16
	Gompertz	99	1155	1168	8
	Weibull	98.6	1210	1225	10

United States	Logistic	98.7	4217	4229	97810
	Exponential	90.6	4546	4559	269037
	Gompertz	99.4	4074	4090	62834
	Weibull	99.6	3999	4015	49898

## Data Availability

The data used to support the findings of this study are available in the Humanitarian Data Exchange at https://data.humdata.org/dataset/novel-coronavirus-2019-ncov-cases?force_layout=desktop.

## Supplementary Materials

Supplementary Materials The supplementary file consists of Figure 1 to Figure 5 which correspond to the separated pre- and post prediction models with intervals for coronavirus disease 2019 infections in Hebei province of China, Italy, Sri Lanka, United States (for the data period from 24^th^ January 2020 to 31^st^ of May 2020), and United States (for the data period from 1^st^ of June 2020 to 2^nd^ of July 2020), respectively.
