# Supplementary material for: Phenomenological Modelling of COVID-19 Epidemics in Sri Lanka, Italy, the United States, and Hebei Province of China
Source: Comput Math Methods Med. 2020 Oct 18;2020:6397063. doi: 10.1155/2020/6397063 (PMC7573661; doi:10.1155/2020/6397063)
Supplement: Supplementary Materials — The supplementary file consists of Figure 1 to Figure 5 which correspond to the separated pre- and post prediction models with intervals for coronavirus disease 2019 infections in Hebei province of China, Italy, Sri Lanka, United States (for the data period from 24th January 2020 to 31st of May 2020), and United States (for the data period from 1st of June 2020 to 2nd of July 2020), respectively. [file 6397063.f1.pdf]

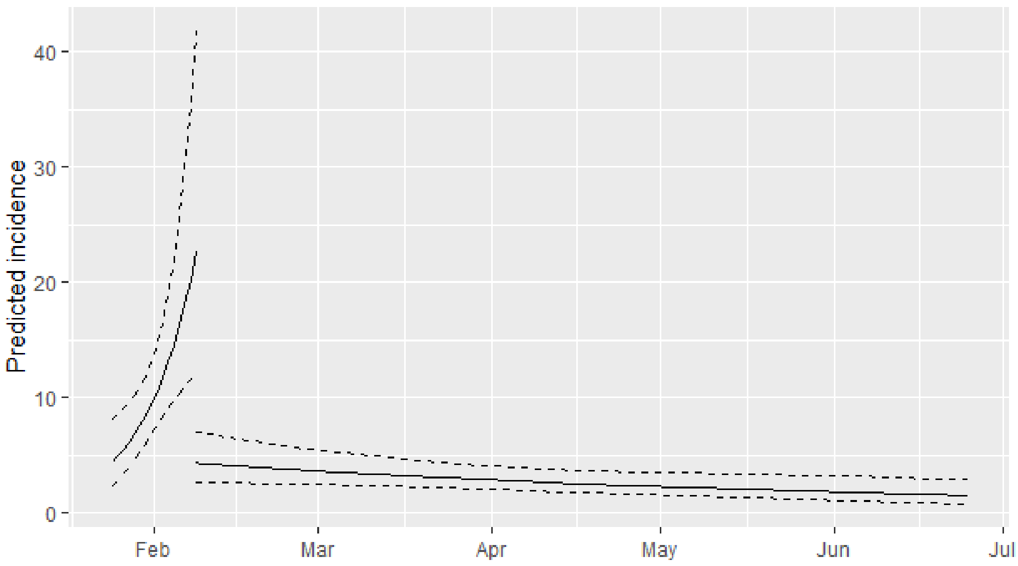

Figure 1: Fitted Prediction Models with Intervals for Infections in Hebei Province, China

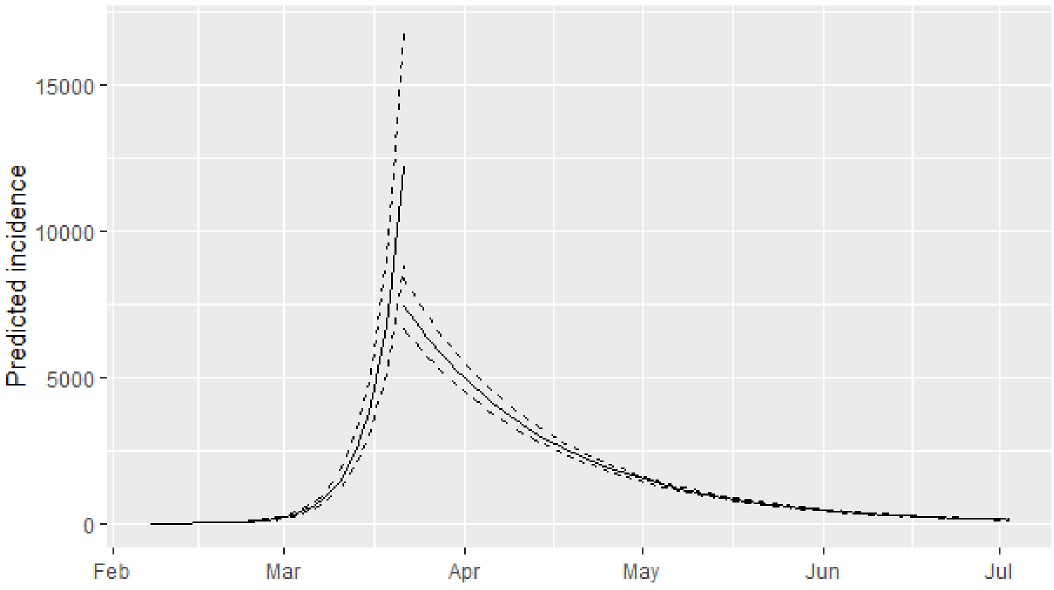

Figure 2: Fitted Prediction Models with Intervals for Infections in Italy

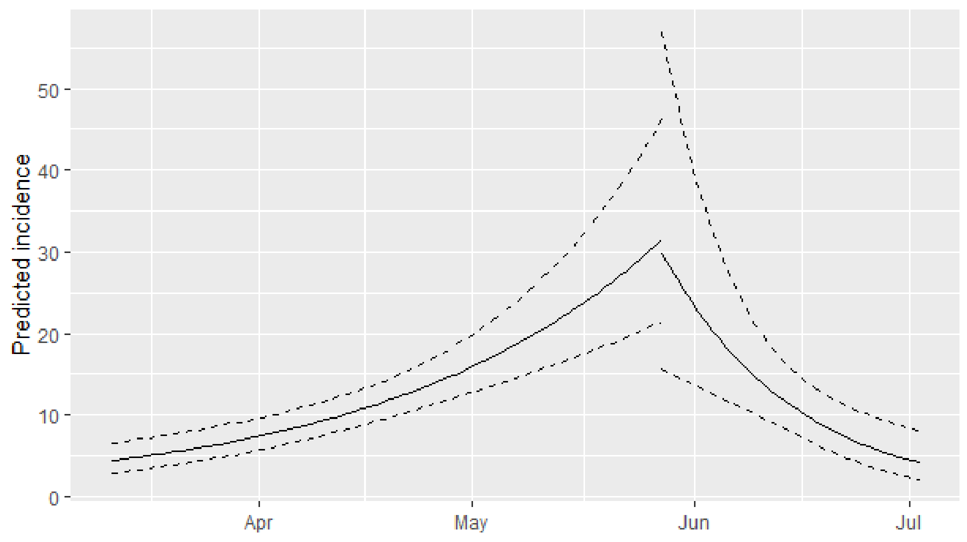

Figure 3: Fitted Prediction Models with Intervals for Infections in Sri Lanka

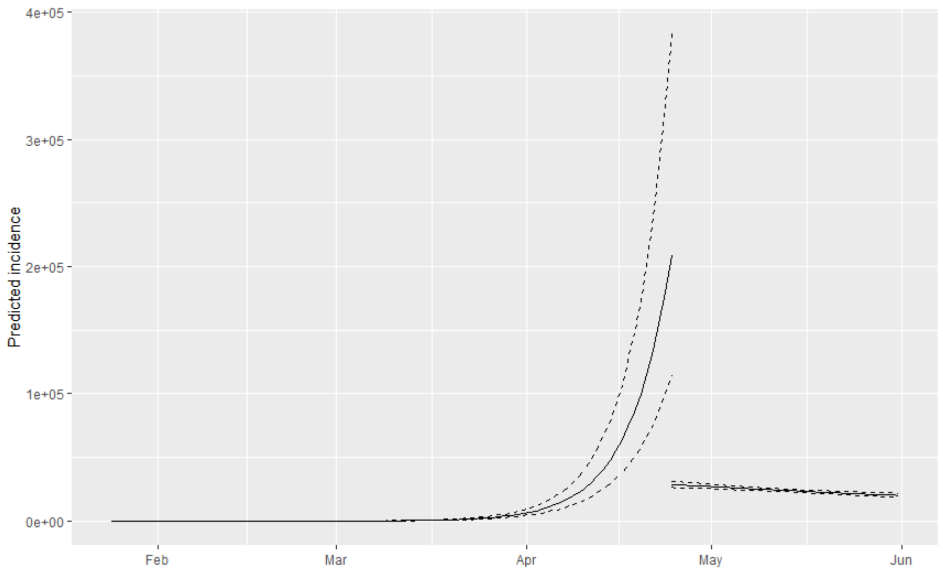

Figure 4: Fitted Prediction Models with Intervals for Infections in United States – First Data Set

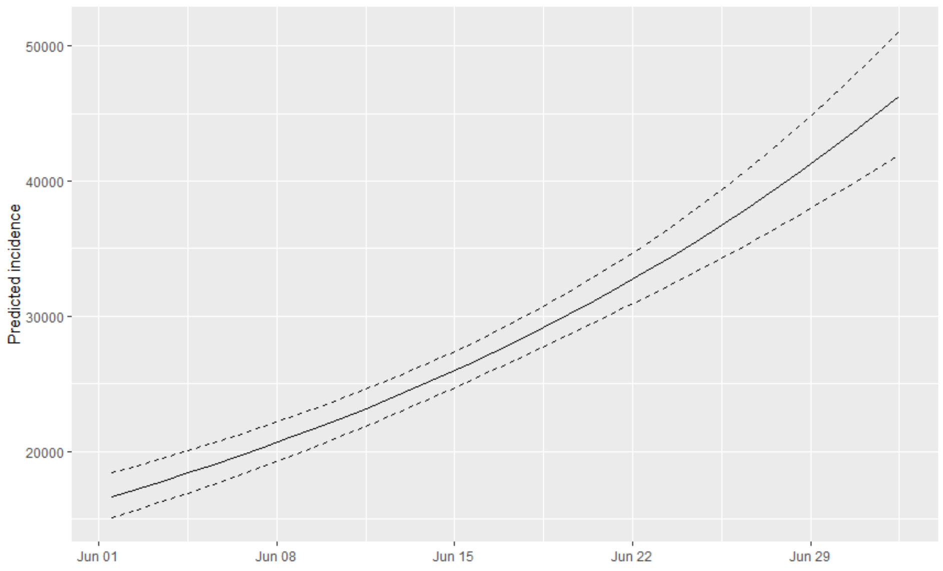

Figure 5: Fitted Prediction Model with Interval for Infections in United States – Second Data Set
